# Comparing Post-Exercise Hypotension after Different Sprint Interval Training Protocols in a Matched Sample of Younger and Older Adults

**DOI:** 10.3390/jcm12020640

**Published:** 2023-01-13

**Authors:** Sascha Ketelhut, Martin Möhle, Tina Gürlich, Laura Hottenrott, Kuno Hottenrott

**Affiliations:** 1 Institute of Sport Science, University of Bern, 3012 Bern, Switzerland; 2 Institute of Sport Science, Martin-Luther-University Halle-Wittenberg, 06120 Halle (Saale), Germany; 3 Institute of Performance Diagnostics and Health Promotion, Martin-Luther-University Halle-Wittenberg, 06120 Halle (Saale), Germany

**Keywords:** post-exercise hypotension, blood pressure, high-intensity interval training, aging

## Abstract

This study assessed the post-exercise hypotension (PEH) effect in a sample of matched young and older adults after different sprint interval training (SIT) protocols. From forty-three participants enrolled in this study, twelve younger (24 ± 3 years) and 12 older (50 ± 7 years) participants, matched for the body mass index, systolic blood pressure, and VO_2_max-percentiles, were selected. The participants completed two SIT protocols consisting of 4 × 30 s exercise bouts interspersed by either one (SIT1) or three minutes (SIT3) of active rest. The peripheral systolic (pSBP) and diastolic (pDBP) blood pressure, central systolic (cSBP) and diastolic (cDBP) blood pressure, pulse wave velocity (PWV), and heart rate (HR) were obtained before and at different measurement time points (t5, t15, t30, t45) after the exercise. No significant time × group interactions were detected in pSBP (*p* = 0.242, η² = 0.060), pDBP (*p* = 0.379, η² = 0.046), cSBP (*p* = 0.091, η² = 0.861), cDBP (*p* = 0.625, η² = 0.033), PWV (*p* = 0.133, η² = 0.076), and HR (*p* = 0.190, η² = 0.123) after SIT1. For SIT3 no significant time × group interactions could be detected for pSBP (*p* = 0.773, η² = 0.020), pDBP (*p* = 0.972, η² = 0.006), cSBP (*p* = 0.239, η² = 0.060), cDBP (*p* = 0.535, η² = 0.036), PWV (*p* = 0.402, η² = 0.044), and HR (*p* = 0.933, η² = 0.009). Matched samples of young and older adults reveal similar PEH effects after HIIT. Accordingly, age does not seem to affect PEH after SIT. These results show that rest interval length and age modulate the PEH effect after SIT.

## 1. Introduction

A bout of exercise can induce a transient reduction in blood pressure [1,2,3]. This phenomenon, known as post-exercise hypotension (PEH), has been proposed to predict long-term changes in blood pressure, due to chronic exercise training [4,5] and thus is respected as a marker for evaluating the blood pressure-related training efficacy [6].

Even though the phenomenon of PEH has been well established after different exercise protocols, the magnitude of the PEH effect varies a lot among studies [3]. Apart from typical moderate-intensity endurance exercises [7], PEH has been reported after intermittent exercise protocols, such as high-intensity interval training (HIIT) [8]. According to a current review, HIIT can induce a more substantial PEH effect than moderate-intensity continuous exercise (MICE) [9].

The efficacy of high-intensity intermittent exercise protocols, concerning cardiorespiratory adaptations, has recently led to greater research on the effects of even shorter and more intense interval protocols, such as sprint interval training (SIT). SIT is structured as four to six 20–45 s all-out sprint efforts interspersed with 1–4.5-min recovery periods of passive or light-intensity exercise [10]. Especially the time-efficient aspect of SIT, compared to MICE or even HIIT, is of interest to practitioners and scholars as the lack of time is commonly cited as a reason for physical inactivity [11,12]. Although the literature addressing PEH in SIT is still sparse, previous studies have shown that SIT can induce a similar PEH effect as HIIT, despite a shorter exercise time [13,14]. However, other studies report conflicting results [15,16,17]. The contradicting results may be explained by the inconsistent exercise protocols applied. A unique feature of SIT is the ability to manipulate the training variables (exercise bout intensity and duration, rest interval intensity and duration, number of exercise bouts, number of series, between-series recovery duration and intensity), which results in various protocols. Surprisingly, few efforts have been made to identify the optimal SIT protocol design [18]. Initial studies show that manipulating training variables, such as the number of sprint bouts [18] or the sprint-to-rest ratio [17,19,20], affects the magnitude of PEH after SIT. Therefore, further research comparing the different SIT protocols is highly warranted to detect the most effective protocols to induce PEH.

Apart from different exercise protocols, the characteristics of the study population may influence the magnitude and duration of the PEH effect [4,21,22]. Especially age [23,24,25], sex [26,27], training status [23,28], and cardiovascular risk factors [23,29], have been reported as influencing factors for PEH and may thus be responsible for the conflicting results.

Especially aging leads to structural and functional changes in the cardiovascular system [30], that can limit the response to vasoactive substances after exercise. Thus, it can be hypothesized that age influences the magnitude of PEH. Surprisingly, previous research has not directly compared the PEH effect between older and younger individuals.

To further our understanding of the PEH effect and its modulating factors, this study aimed to compare the PEH effect in a matched sample of older and younger adults after two different SIT protocols. Since the body mass index (BMI), endurance performance, sex, and blood pressure have been shown to affect PEH [23,31,32], the groups were matched for these confounding factors. We hypothesize that both age and the protocol design modulate the magnitude of the PEH effect after SIT.

## 2. Materials and Methods

### 2.1. Participants and the Study Design

A randomized crossover study design was implemented involving a sample of healthy individuals. Participants were eligible for the study if they were (1) 18 years or older, (2) participated in regular strenuous endurance exercise, and >6 h/week of cycling training for at least six months prior to the study, (3) had no underlying health condition that could compromise the safety of the physical exercise, and (4) were not under cardiovascular medications. Participants were recruited via personal contacts, social media posts, and local sports clubs.

Forty-three participants volunteered to participate in this trial. The participants reported to the lab of the Institute of Sport Science of the Martin-Luther-University Halle-Wittenberg for a baseline examination. All participants gave informed consent. The study was conducted in accordance with the Declaration of Helsinki, and approved by the institutional ethics committee of the Medical Faculty of the Martin-Luther-University Halle-Wittenberg (2019-094, 12 July 2019).

The participants were instructed to be at least two hours postprandial, abstain from caffeine and alcohol for at least four hours, and avoid exercising for at least 12 h prior to testing. During the visit, the demographic and anthropometric data were obtained. Furthermore, different hemodynamic parameters, height, and weight were measured, and a standard graded cardiopulmonary exercise stress was conducted on a bicycle ergometer (E 2000 s, FES, Berlin, Germany). Following the baseline examination, 12 younger (24 ± 3 years, 50% female) and 12 older (50 ± 7 years, 50% female) participants, matched for the BMI, peripheral systolic blood pressure (pSBP), and maximum oxygen consumption (VO_2_max) percentiles (Graves et al., 2015) were selected for further analysis.

**Figure 1:**
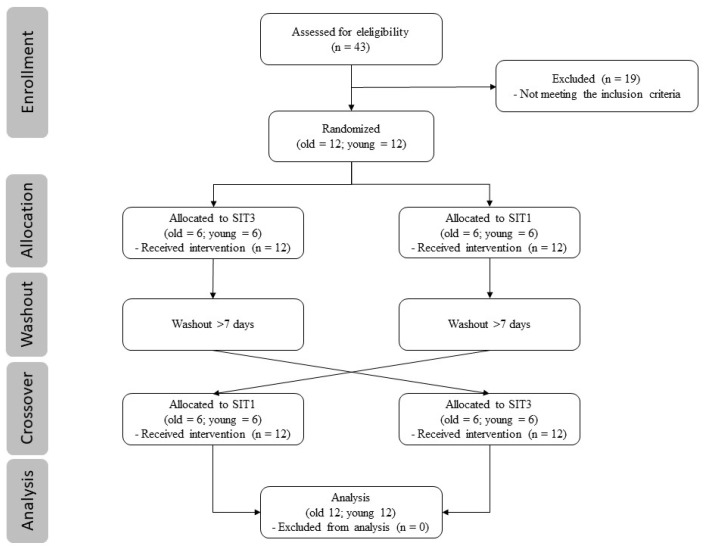
Flow diagram.

The selected participants completed two different SIT protocols on a bicycle ergometer (E 2000 s, FES, Berlin, Germany) in a randomized and balanced order on the second and third visits. To avoid the circadian influence on the outcomes, all visits occurred in the morning, at the same time. Between the three visits, at least seven but not more than 14 days were allowed (Figure 1). Until the completion of all test days, the participants were requested to maintain their usual diet.

### 2.2. Baseline Examination

Demographic data, medical history, and habitual physical activity were assessed using questionnaires (Freiburger Physical Activity Questionnaire [Freiburger Fragebogen zur körperlichen Aktivität] [33]). The standing height and body mass were assessed with participants wearing light clothing, using a wall-mounted stadiometer and scale (BC-545N, Tanita Europe BV, Netherlands). Thereafter the hemodynamic parameters were assessed after a 10-min supine rest (Mobil-O-Graph, IEM, Stolberg Germany). The participants then completed a graded exercise test on a high-performance bicycle ergometer (E 2000 S, FES, Berlin, Germany). The test started with an eight-minute warm-up at 100 watts (males) or 70 watts (females). Following the warm-up, the test started with an initial workload of 70 watts. Every minute thereafter, the workload increased by 30 watts until volitional fatigue, determined by the failure to maintain a cycle cadence of 60 revolutions per minute (RPM). The cadence was set at 80–90 RPM. To determine whether VO_2_max was attained, three of the following five criteria had to be met: (1) a final rating of perceived exertion score of ≥17 on the Borg scale (scale 6–20), (2) no change in the heart rate (HR) with a change in workload, (3) a respiratory exchange ratio >1.1, (4) a “plateau” in the oxygen uptake with an increase in workload, (5) volitional fatigue, defined as an inability to maintain a pedal rate above 60 revolutions per minute. The HR was recorded beat-to-beat throughout the test, using a Polar HR monitor (RS800 CX, Polar Electro GmbH, Helsinki, Finland). Oxygen consumption was collected continuously and analyzed using a breath-by-breath gas collection system (Metalyzer 3B, Cortex, Leipzig, Germany). Participants were verbally encouraged to go to volitional exhaustion. VO_2_max was calculated as the highest recorded value, using the recorded rolling average of 15 s epochs.

### 2.3. Experimental Sessions

**Figure 2:**
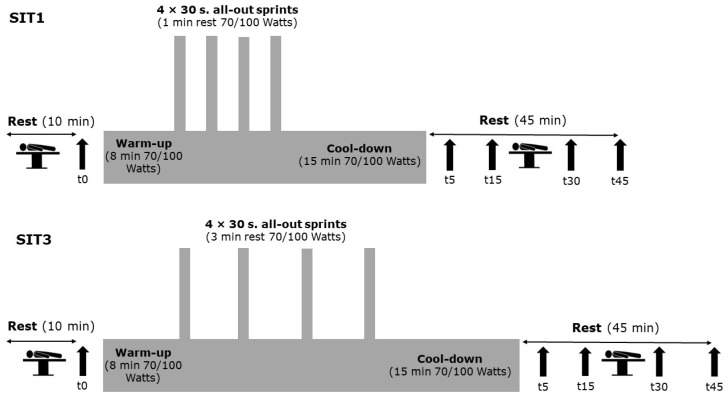
Protocol design overview for the sprint interval training with 1 min rest (SIT1) and 3 min rest (SIT3) between bouts. t0 = rest, t5 = 5 min after exercise, t15 = 15 min after exercise, t30 = 30 min after exercise, t45 = 45 min after exercise.

The participants performed two experimental sessions in a random and balanced order (Figure 2). Each session started with a 10-min supine rest followed by two blood pressure readings. The mean of the two readings was further analyzed. Thereafter, all participants completed an 8-min warm-up at 70 watts for the female and 100 watts for the male participants. The cadence was set at 80–90 revolutions per minute. The following SIT protocols consisted of 4 × 30 s all-out cycling sprint bouts separated by either 1 min (SIT1) or 3 min (SIT3) of active recovery (70 watts female, 100 watts male). The protocol ended with a 15-min cool-down at 70 watts for the female and 100 watts for the male participants. Throughout the 45 min after the training, the hemodynamic parameters and the HR were obtained.

### 2.4. Hemodyanmic Parameters

The peripheral systolic (pSBP) and diastolic (pDBP) blood pressure, the central systolic (cSBP) and diastolic (cDBP) blood pressure, and the pulse wave velocity (PWV) were assessed using the Mobil-O-Graph (24 PWA monitor, IEM, Stolberg Germany). All readings were executed by the same study staff member under controlled conditions. The readings were obtained in the supine position, following 10 min of rest before exercise, and 5, 15, 30, and 45 min postexercise. A properly sized cuff was placed on the right arm about 2 cm above the antecubital fossa.

### 2.5. Sample Size and Randomization

An a priori power analysis using G*Power (Version 3.1.2; Heinrich Heine Universität, Dusseldorf, Germany) was conducted. Assuming an effect size of 0.25 [32] with an alpha level of 0.05, 20 participants would be necessary for an appropriate power (0.8). The randomization process was conducted by the principal investigator using a computer-generated random number table, stratified for pSBP, BMI, and VO_2_max percentiles.

### 2.6. Statistics

All statistical analyses were performed using IBM SPSS Statistics v. 27.0 (SPSS, Chicago, IL, USA). The continuous variables are expressed as means ± standard deviation. Student’s *t*-tests were performed to determine the possible differences in the subject characteristics at the baseline. The repeated measures ANOVA with the Bonferroni corrections for multiple comparisons, if warranted, was used to investigate the effect of the group (older versus young) over time (t0 and t5, t15, t30, t45-min post-exercise) on the outcomes. Mauchly’s test was used to investigate the sphericity of the interaction term, with a Greenhouse–Geisser correction applied if the assumption of sphericity was violated. Where appropriate, post hoc analyses, including the one-way ANOVA, were performed with Bonferroni’s correction. A *p*-value < 0.05 was considered statistically significant. Partial eta-squared (η^2^) values were calculated to estimate the effect sizes (small effect: 0.04, medium effect: 0.06, large effect: 0.14) for the interactions.

## 3. Results

No adverse events were documented in any of the participants during the exercise sessions. The baseline characteristics of both groups are summarized in Table 1. Apart from age, there were significant differences in pDBP, cDBP, and PWV between the groups, with the older participants showing higher values. According to the classification of the European Society of Cardiology [34], one of the younger and two of the older participants had high normal blood pressure. Based on the BMI, three older participants and one of the younger participants were classified as slightly overweight [35]. Regarding the VO_2_max-percentiles, both groups could be classified as highly trained. All participants reported being engaged in regular cycling exercises for more than six weeks. Apart from that, they reported participating in running, swimming, triathlon, or cycling exercises with a training volume of 8.33 ± 2.8 h per week.

### 3.1. SIT1

#### 3.1.1. Peripheral Blood Pressure

Following SIT1, no significant time × group interactions were detected in the pSBP (F(4,88) = 1.39, *p* = 0.242, η² = 0.060), and the pDBP (F(4,88) = 1.07, *p* = 0.379, η² = 0.046). A significant time effect (F(4,88) = 2.86, *p* = 0.028, η² = 0.115), and a non-significant group effect (F(1,22) = 0.06, *p* = 0.803, η² = 0.003) was detected in the pSBP. For the pDBP, a significant time (F(4,88) = 8.55, *p* < 0.001, η² = 0.280), and group effect (F(1,22) = 7.05, *p* = 0.014, η² = 0.243) were observed.

#### 3.1.2. Central Blood Pressure

No significant time × group interactions were detected in the cSBP (F(4,88) = 2.08, *p* = 0.091, η² = 0.861) and the cDBP (f(4,88) = 0.66, *p* = 0.625, η² = 0.033). There was a significant main effect for time (F(4,88) = 7.55, *p* < 0.001, η² = 0.255), but not for group (F(1,22) = 0.87, *p* < 0.360, η² = 0.038) in the cSBP. In the cDBP, a significant time effect (F(4,88) = 9,71, *p* < 0.001, η² = 0.316) and a significant group effect (F(1,22) = 5.85, *p* = 0.025, η² = 0.218) were detected.

#### 3.1.3. Pulse Wave Velocity and the Heart Rate

In the PWV, there was no significant interaction effect (F(4,88) = 1.81, *p* = 0.133, η² = 0.076). Again, significant main effects for time (F(4,88) = 6.28, *p* < 0.001, η² = 0.222) and group (F(1,22) = 44.74, *p* < 0.001, η² = 0.670) were found. For changes in the HR, no significant interaction effect was detected (F(4,88) = 3.10, *p* = 0.190, η² = 0.123). Significant main effects for time (F(4,88) = 75.67 *p* < 0.001, η² = 0.775) but not group (F(1,22) = 0.08, *p* = 0.703, η² = 0.004) were observed.

In the young, there was a significant increase in the pDBP (*p* = 0.013) and the cDBP at t5 (*p* = 0.022), compared to the baseline. The older revealed a significant increase in the HR at t5 (*p* < 0.001) and t15 (*p* < 0.001), compared to the rest (Table 2).

### 3.2. SIT3

#### 3.2.1. Peripheral Blood Pressure

No significant time × group interactions could be detected in the pSBP (F(4,88) = 0.45, *p* = 0.773, η² = 0.020), and the pDBP (F(4,88) = 0.13, *p* = 0.972, η² = 0.006). There was a significant main effect for time (F(4,88) = 47.54, *p* < 0.001, η² = 0.684), but not for group (F(1,22) = 0.168, *p* < 0.686, η² = 0.008) in the pSBP. A significant main effects for time (F(4,22) = 13.52, *p* < 0.001, η² = 0.381), and group (F(1,22) = 9.88, *p* = 0.005, η² = 0.310) was detected for change in the pDBP.

#### 3.2.2. Central Blood Pressure

No significant time × group interaction could be detected in the cSBP (F(4,88) = 1.40, *p* = 0.239, η² = 0.060), with a significant effect for time (F(4,88) = 32.08, *p* < 0.001, η² = 0.593), but not for group (F(1,22) = 0.93, *p = 0.346,* η² = 0.041). No significant time × group interaction could be detected in the cDBP (F(4,88) = 0.79, *p* = 0.535, η² = 0.036), with a significant main effect for time (F(4,88) = 14.66, *p* < 0.001, η² = 0.411), and group (F(1,21) = 8.82, *p* = 0.007, η² = 0.296).

#### 3.2.3. Pulse Wave Velocity and Heart Rate

No significant time × group interaction could be detected in the PWV (F(4,88) = 1.02, *p* = 0.402, η² = 0.044), with a significant main effect for time (F(4,88) = 14.69, *p* < 0.001, η² = 0.400), and a significant main effect for group (F(1,22) = 50.83, *p* < 0.001, η² = 0.698). No significant time × group interaction could be detected in the HR (F(4,88) = 0.21, *p* = 0.933, η² = 0.009). There was a significant main effect for time (F(4,88) = 37.32, *p* < 0.001, η² = 0.629), but not for group (F(1,22) = 0.10, *p* < 0.753, η² = 0.005).

Compared to the resting value, the pSBP was significantly lower at t15 (*p* = 0.001), t30 (*p* < 0.001), and t45 (*p* < 0.001) in the older and at t30 (*p* = 0.008), and t45 (*p* = 0.001) in young individuals. Compared to the baseline, the pDBP was significantly lower at t30 (*p* = 0.017), and the cSBP was lower at t30 (*p* = 0.018) and t45 (*p* = 0.002) in the older participants. Regarding the cDBP, only the older revealed a significant reduction at t30 (*p* = 0.006). Similarly, the PWV was reduced only in the older at t45 (*p* = 0.003). Both groups showed a significant increase in the HR at t5 (old: *p* < 0.001; young: *p* = 0.005). In the young, the HR was still significantly increased at t15 (*p* = 0.010), t30 (*p* = 0.010), and t45 (*p* = 0.025) (Table 2).

## 4. Discussion

This study aimed to compare the PEH effects after two different SIT protocols in a sample of matched young and older adults. The results show that, if matched for the BMI, systolic blood pressure, sex, and VO_2_max-percentiles, older and younger adults experience similar PEH effects after SIT3. Both groups showed no PEH effects after the shorter SIT1.

The reduction in the peripheral blood pressure after SIT3 is in line with previous studies assessing the acute responses to different SIT protocols consisting of four to six bouts of 30 s all-out sprints interspersed by 4–4.5 min of rest [13,14,16]. Only very few studies addressed the PEH effect after the SIT protocols with shorter rest intervals (32–60 s), reporting inconsistent results [17,20].

Interestingly previous studies detected considerably lower mean blood pressure reductions after SIT, compared to SIT3 in the present study. In the study by Angadi et al. [13], the reduction in the systolic blood pressure was only 1 mmHg. A more substantial reduction was detected by Rossow et al. [14], who found a reduction of 5 mmHg in the systolic blood pressure following SIT consisting of 4 × 30 s bouts with 4.5 min active recovery. In the present study, the systolic blood pressure decreased by 13 mmHg in the old and 11 mmHg in the young.

Unfortunately, research predominantly focused on the relatively young adults (<30 years). This is the first study to report the PEH effects after SIT in older adults. The magnitude of PEH after SIT3 is similar to the previous research on HIIT, MICE, and resistance training [9,36,37]. Thus, SIT3 seems to represent a time-efficient exercise protocol that can trigger a relevant PEH effect in younger and older adults.

The fact that there were no significant differences between the younger and older participants in the current study conflicts with a previous review by Carpio-Rivera et al. [23], observing an inverse association between age and the magnitude of PEH. The authors [23] speculate that age-related structural and functional changes in the vascular system limit the response to circulating vasodilators during exercise [38], blunting the PEH effect. This is supported by a review from Brito et al. [31], reporting that PEH in younger adults seems to be primarily attributed to a reduction in the peripheral vascular resistance, while a reduction in the cardiac output seems to be the dominant mechanism in older adults. In the included studies, the review by Brito et al. [31] determined a decrease in peripheral resistance in 74% of cases involving young subjects and in 62% of cases involving middle-aged subjects. For the elderly, no changes in the peripheral resistance could be observed. However, in this group, the cardiac output was reduced in 75% of cases. This is supported by Rondon et al. [39], reporting a significant reduction in the cardiac output and stroke volume in 24 elderly patients after MICE. In contrast to Carpio-Rivera et al. [23], Brito et al. [31] conclude that PEH is not blunted with age, however, there seems to be different underlying mechanisms in younger and older adults.

Unfortunately, the present study has not obtained the peripheral resistance or cardiac output. However, the PWV was assessed, reflecting the arterial stiffness, and is therefore regarded as a marker for the structural vascular alterations [40]. Interestingly, a significant reduction in the PWV could only be detected in the older participants after SIT3. This finding contradicts the assumption that the responsiveness to the vasodilator agents is altered with age.

Differences in the exercise protocols may explain the conflicting results. The reviews by Brito et al. [31] and Carpio-Rivera et al. [23] included studies investigating various exercise protocols (interval training and continuous exercise) but not SIT. Thus, it is assumed that the exercise intensity in the current study was considerably higher, as the protocol required the participants to perform all-out sprints. Previous research shows that a higher exercise intensity leads to a higher vascular shear stress and, therefore, a stronger nitric oxide release, which results in a lower total peripheral resistance [14,41]. Furthermore, stronger fluctuations in the cardiac output can be discussed. The intermittent character of the SIT protocol and the high exercise intensity, paired with the relatively long rest intervals may result in stronger blood flow variations than during MICE or even HIIT. Blood flow variation has been discussed as a trigger for the pulsate and shear stress and, thus, nitric oxide release [16].

Apart from the differences in the exercise protocols, the fact that the studies included in the reviews did not consider the participants’ characteristics, could be an explanation for the conflicting results. Thus, the PEH effect, and the underlying mechanism, may have been masked by differences in the confounding factors, such as training status/physical fitness, resting blood pressure, and the BMI. To isolate the age-specific effect, it is crucial to match the groups for these confounding factors.

Our study represents the first direct comparison between matched samples of older and younger adults. Based on the results, it may be assumed that it is not the age perse that affects PEH but rather age-related confounders. It has previously been reported that the BMI [42], resting blood pressure [31,43], and physical fitness [23,28] modulate PEH. As these are all factors influenced by lifestyle changes [44], previous studies may have only measured the lifestyle changes often related to aging, but not direct age-specific differences.

Apart from the peripheral blood pressure, the present study is one of the first to also assess the acute effects of SIT on the central blood pressure, showing a significant reduction 45 min after SIT3 in the elderly. The prognostic value of the central blood pressure is higher than that of the peripheral BP as it reflects the afterload of the heart and correlates with the myocardial oxygen consumption [45]. Only Rossow et al. [14] have previously assessed the effects of SIT on the central blood pressure and found a significant decrease after SIT in younger individuals. The same has been shown for HIIT consisting of 6 × 1 min exercise bouts with 4 min of rest between the bouts [8].

Interestingly, no PEH effect could be detected after SIT1 in either of the groups. As stated earlier, this is in line with previous research [17,20] and may be attributed to a higher total exercise volume [23] or higher exercise intensities [20] in SIT3, compared to SIT1. Furthermore, it could be debated that the longer rest periods during SIT3 resulted in a stronger reduction in stroke volume between the exercise bouts, consequently leading to higher fluctuations in the cardiac output and thus the greater blood flow variations leading to the more pronounced NO release [16].

Although the underlying physiological mechanisms responsible for the present results are not clear, it can be concluded that the length of the rest interval and age are factors that modulate PEH after SIT. Based on the present results, SIT3 appears to provide a stronger physiological stimulus, compared to SIT1. These results provide important information for the prescription of SIT. However, further studies are needed to investigate the effects of other SIT protocols, to determine the optimal protocol. The fact that the PEH effect predicts the long-term changes in the blood pressure due to chronic exercise training, underlines the relevance of the present results [5].

### Limitations

Some limitations should be taken into account when interpreting the present results. First, the generalizability of the findings is limited by the normotensive and physically fit population, as well as the specific SIT protocols applied. Further studies are warranted, assessing the hemodynamic effects of different SIT protocols in inactive and maybe at risk patients.

Second, we only assessed the PEH effect throughout 45 min of recovery. It would be interesting to monitor the hemodynamic parameters over an extended period.

A third limitation is the fact that females were not tested during a standardized menstrual cycle phase. Previous research on the influence of the menstrual cycle phase on PEH reports conflicting results [46]. It cannot be ruled out that differences in the hormone status among our female participants could have influenced the results.

Fourth, it would be interesting to assess the additional cardiovascular parameters (cardiac output, peripheral resistance, etc.) to better determine the mechanisms responsible for the PEH effect and to identify the possible differences between the young and older people.

Fifth, apart from determining the health-related benefits, it would have been interesting to assess the exercise enjoyment to determine if SIT protocols have the potential for long-term maintenance [47].

## 5. Conclusions

This is the first study to compare the PEH effects after different SIT protocols in a sample of matched older and younger adults. The results show that SIT3 triggers a PEH effect in younger and older adults and may thus represent an effective alternative to MICE or HIIT. Contrary to the common assumption, aging does not perse affect PEH. Rather, age-related lifestyle factors could be the modulators for the differences reported in previous research. Thus, it is crucial to consider these confounding factors in studies addressing PEH. Future studies addressing further cardiovascular parameters are warranted to detect possible differences in the underlying mechanisms of the PEH effect in older and younger individuals. Furthermore, different exercise protocols should be applied, as it seems that manipulating the training variables influences PEH.

## Figures and Tables

**Table 1:** Participant’s characteristics.

Outcome	Total	Young	Older	*p*-Values
Gender (f/m)	12/12	6/6	6/6	
Age (years)	37 ± 14	24 ± 3	50 ± 7	<0.001 ***
Body mass (kg)	66.9 ± 9.4	66.4 ± 8.8	67.4 ± 10.4	0.792
Height (cm)	173.4 ± 10.1	176.4 ± 11.0	170.3 ± 8.4	0.141
Body-mass-index (kg/m^2^)	22.4 ± 2.7	21.4 ± 1.9	23.5 ± 3.1	0.056
VO_2_max (mL/kg/min)	47.8 ± 8.8	50.6 ± 9.2	45.0 ± 7.7	0.111
VO_2_max-percentile	85.2 ± 12.5	84.8 ± 12.4	85.6 ± 13.1	0.884
Training h/week	8.33 ± 2.8	8.3 ± 2.6	8.3 ± 3.1	0.999
pSBP (mmHg)	119 ± 12	119 ± 13	120 ± 11	0.786
pDBD (mmHg)	73 ± 9	69 ± 8	77 ± 8	0.010 *
cSBP (mmHg)	110 ± 13	107 ± 14	112 ± 11	0.294
cDBP (mmHg)	74 ± 8	70 ± 8	78 ± 7	0.019
PWV (m/s)	6.1 ± 1.3	5.1 ± 0.5	7.1 ± 0.9	<0.001 ***
HR (min^−1^)	59 ± 10	57 ± 11	61 ± 8	0.244

Data expressed as the mean ± standard deviations. *p*-values indicate the differences between the young and old. VO_2_max = maximal oxygen consumption, VO_2_max-percentile = maximal oxygen consumption percentile curves (Graves et al., 2015), pSBP = peripheral systolic blood pressure, pDBP = peripheral diastolic blood pressure, cSBP = central systolic blood pressure, cDBP = central diastolic blood pressure, PWV = pulse wave velocity, HR = heart rate. * *p* < 0.05, *** *p* < 0.001, difference between groups.

**Table 2:** Hemodynamic parameters at rest before (rest) and 5, 15, 30, and 45 min after the two sprint interval training protocols in groups of younger (Y) and older (O) adults.

Outcome	Protocol	Group	rest	5 min.	15 min.	30 min.	45 min.	*p*-Values (Group × Time)
pSBP (mmHg)	SIT1	O	120 ± 12	121 ± 10	119 ± 10	120 ± 12	119 ± 13	0.242
		Y	119 ± 13	123 ± 12	119 ± 12	118 ± 12	115 ± 11	
pDBP (mmHg)		O	78 ± 8	81 ± 7	80 ± 7	78 ± 6	77 ± 6	0.373
		Y	69 ± 8	75 ± 11 *	72 ± 10	69 ± 11	66 ± 9	
cSBP (mmHg)		O	112 ± 11	117 ± 10	115 ± 10	115 ± 13	114 ± 12	0.091
		Y	107 ± 14	116 ± 12	114 ± 11	111 ± 10	105 ± 11	
cDBP (mmHg)		O	78 ± 7	82 ± 8	81 ± 8	78 ± 6	77 ± 7	0.625
		Y	70 ± 8	76 ± 11 *	73 ± 11	69 ± 11	68 ± 9	
PWV (m/s)		O	7.1 ± 0.9	7.2 ± 0.9	7.2 ± 0.8	7.2 ± 0.8	7.1 ± 0.9	0.133
		Y	5.1 ± 0.5	5.4 ± 0.5	5.3 ± 0.4	5.3 ± 0.4	5.1 ± 0.4	
HR (min^−1^)		O	61 ± 8	78 ± 8 ***	75 ± 8 ***	65 ± 5	63 ± 6	0.190
		Y	57 ± 11	82 ± 12	76 ± 12	68 ± 13	64 ± 12	
pSBP (mmHg)	SIT3	O	124 ± 7	124 ± 10	116 ± 8 **	116 ± 8 ***	111.5 ± 8 ***	0.773
		Y	121 ± 12	122 ± 10	116 ± 10	114 ± 8 **	110 ± 10 ***	
pDBP (mmHg)		O	79 ± 6	82 ± 7	79 ± 5	75 ± 6 *	76 ± 7	0.972
		Y	70 ± 9	73 ± 7	71 ± 8	67 ± 8	67 ± 10	
cSBP (mmHg)		O	116 ± 7	119 ± 10	113 ± 9	110 ± 9 *	107 ± 7 **	0.239
		Y	110 ± 10	118 ± 9 *	110 ± 9	107 ± 7	104 ± 9	
cDBP (mmHg)		O	80 ± 6	83 ± 7 *	80 ± 5	75 ± 6 **	76 ± 7	0.535
		Y	71 ± 9	74 ± 8	72 ± 8	68 ± 8	69 ± 10	
PWV (m/s)		O	7.2 ± 0.8	7.3 ± 0.9	7.1 ± 0.8	7.0 ± 0.7	6.9 ± 0.8 **	0.402
		Y	5.2 ± 0.4	5.5 ± 0.6	5.3 ± 0.4	5.2 ± 0.4	5.1 ± 0.4	
HR (min^−1^)		O	62 ± 8	81 ± 7 ***	73 ± 8	69 ± 6	66 ± 7	0.933
		Y	58 ± 9	81 ± 15 **	73 ± 12 *	68 ± 10 *	66 ± 10 *	

Data are presented as mean ± standard deviation. pSBP = peripheral systolic blood pressure, pDBP = peripheral diastolic blood pressure; cSBP = central systolic blood pressure; cDBP = central diastolic blood pressure; PWV = pulse wave velocity; HR = heart rate. SIT1 = sprint interval training with 1-min rest between bouts, SIT3 = sprint interval training with 3 min rest between bouts. *p*-values indicate interaction effects. * *p* < 0.05, ** *p* < 0.01, *** *p* < 0.001, changes from values at rest.

## Data Availability

The data presented in this study are available upon request from the corresponding author.

## References

[1] Halliwill J.R. (2001). Mechanisms and Clinical Implications of Post-exercise Hypotension in Humans. Exerc. Sport Sci. Rev..

[2] Halliwill J.R., Buck T.M., Lacewell A.N., Romero S.A. (2013). Postexercise hypotension and sustained postexercise vasodilatation: What happens after we exercise?. Exp. Physiol..

[3] Pescatello L.S., Franklin B.A., Fagard R., Farquhar W.B., Kelley G.A., Ray C.A. (2004). Exercise and Hypertension. Med. Sci. Sport. Exerc..

[4] Brito L.C., Fecchio R.Y., Peçanha T., Andrade-Lima A., Halliwill J.R., Forjaz C.L.M. (2018). Postexercise hypotension as a clinical tool: A “single brick” in the wall. J. Am. Soc. Hypertens..

[5] Wegmann M., Hecksteden A., Poppendieck W., Steffen A., Kraushaar J., Morsch A., Meyer T. (2018). Postexercise Hypotension as a Predictor for Long-Term Training-Induced Blood Pressure Reduction. Clin. J. Sport Med..

[6] Hecksteden A., Grütters T., Meyer T. (2013). Association Between Postexercise Hypotension and Long-term Training-Induced Blood Pressure Reduction. Clin. J. Sport Med..

[7] Milatz F., Ketelhut S., Ketelhut R.G. (2015). Favorable effect of aerobic exercise on arterial pressure and aortic pulse wave velocity during stress testing. Vasa.

[8] Ketelhut S., Milatz F., Heise W., Ketelhut R.G. (2016). Influence of a high-intensity interval training session on peripheral and central blood pressure at rest and during stress testing in healthy individuals. Vasa.

[9] Perrier-Melo R.J., Costa E.C., Farah B.Q., Costa M.d.C. (2020). Acute effect of interval vs. Continuous exercise on blood pressure: Systematic review and meta-analysis. Arq. Bras. Cardiol..

[10] Laursen P., Buchheit M. (2019). Science and Application of High-Intensity Interval Training.

[11] Trost S.G., Pate R.R., Sallis J.F., Freedson P.S., Taylor W.C., Dowda M., Sirard J. (2002). Age and gender differences in objectively measured physical activity in youth. Med. Sci. Sport. Exerc..

[12] Korkiakangas E.E., Alahuhta M.A., Laitinen J.H. (2009). Barriers to regular exercise among adults at high risk or diagnosed with type 2 diabetes: A systematic review. Health Promot. Int..

[13] Angadi S.S., Bhammar D.M., Gaesser G.A. (2015). Postexercise Hypotension after Continuous, Aerobic Interval, and Sprint Interval Exercise. J. Strength Cond. Res..

[14] Rossow L., Yan H., Fahs C.A., Ranadive S.M., Agiovlasitis S., Wilund K.R., Baynard T., Fernhall B. (2010). Postexercise hypotension in an endurance-trained population of men and women following high-intensity interval and steady-state cycling. Am. J. Hypertens..

[15] Chan H.H., Burns S.F. (2013). Oxygen consumption, substrate oxidation, and blood pressure following sprint interval exercise. Appl. Physiol. Nutr. Metab..

[16] Stuckey M.I., Tordi N., Mourot L., Gurr L.J., Rakobowchuk M., Millar P.J., Toth R., Macdonald M.J., Kamath M.V. (2012). Autonomic recovery following sprint interval exercise. Scand. J. Med. Sci. Sport..

[17] Ketelhut S., Möhle M., Gürlich T., Hottenrott L., Hottenrott K. (2022). Optimizing sprint interval exercise for post-exercise hypotension: A randomized crossover trial. Eur. J. Sport Sci..

[18] Vollaard N.B.J., Metcalfe R.S., Williams S. (2017). Effect of number of sprints in an SIT session on change in v O2max: A meta-analysis. Med. Sci. Sport. Exerc..

[19] Boyne P., Dunning K., Carl D., Gerson M., Khoury J., Kissela B. (2014). Within-session responses to high-intensity interval training in chronic stroke. Med. Sci. Sport. Exerc..

[20] Jones M.D., Munir M., Wilkonski A., Ng K., Beynon G., Keech A. (2021). Post-exercise hypotension time-course is influenced by exercise intensity: A randomised trial comparing moderate-intensity, high-intensity, and sprint exercise. J. Hum. Hypertens..

[21] Gomes J.L.d.B., Vancea D.M.M., Cappato de Araújo R., Soltani P., Guimarães F.J.d.S.P., Costa M.d.C. (2021). Cardiovascular and Enjoyment Comparisons after Active Videogame and Running in Type-1 Diabetics: A Randomized Crossover Trial. Games Health J..

[22] Pimenta F.C., Tanil F., Victor M., Dourado Z., Fernando L., Alves G., Wesley B., Vieira D.O., Medeiros A. (2019). High-intensity interval exercise promotes post-exercise hypotension of greater magnitude compared to moderate-intensity continuous exercise. Eur. J. Appl. Physiol..

[23] Carpio-Rivera E., Moncada-Jiménez J., Salazar-Rojas W., Solera-Herrera A. (2016). Acute effects of exercise on blood pressure: A meta-analytic investigation. Arq. Bras. Cardiol..

[24] Nickel K.J., Acree L.S., Gardner A.W. (2011). Effects of a single bout of exercise on arterial compliance in older adults. Angiology.

[25] Harvey P., Morris B.L., Kubo T., Picton P.E., Su W.S., Notarius C.F., Floras J.S. (2005). Hemodynamic after-effects of acute dynamic exercise in sedentary normotensive postmenopausal women. J. Hypertens..

[26] Senitko A.N., Charkoudian N., Halliwill J.R. (2002). Influence of endurance exercise training status and gender on postexercise hypotension. J. Appl. Physiol..

[27] de Oliveira Carpes L., Domingues L.B., Schimitt R., Ferrari R. (2021). Sex Differences in Post-exercise Hypotension, Ambulatory Blood Pressure Variability, and Endothelial Function after a Power Training Session in Older Adults. Front. Physiol..

[28] Iellamo F., Caminiti G., Montano M., Manzi V., Franchini A., Mancuso A., Volterrani M. (2021). Prolonged Post-Exercise Hypotension: Effects of Different Exercise Modalities and Training Statuses in Elderly Patients with Hypertension. Int. J. Environ. Res. Public Health.

[29] Kaufman F.L., Hughson R.L., Schaman J.P. (1987). Effect of exercise on recovery blood pressure in normotensive and hypertensive subjects. Med. Sci. Sport. Exerc..

[30] Lakatta E.G., Levy D. (2003). Arterial and cardiac aging: Major shareholders in cardiovascular disease enterprises: Part II: The aging heart in health: Links to heart disease. Circulation.

[31] Brito L.C., Queiroz A.C.C., Forjaz C.L.M. (2014). Influence of population and exercise protocol characteristics on hemodynamic determinants of post-aerobic exercise hypotension. Braz. J. Med. Biol. Res..

[32] Forjaz C.L.M., Tinucci T., Ortega K.C., Santaella D.F., Mion D., Negrão C.E. (2000). Factors affecting post-exercise hypotension in normotensive and hypertensive humans. Blood Press. Monit..

[33] Frey I., Berg A., Grathwohl D., Keul J. (1999). Freiburger Fragebogen zur kSrperlichen Aktivit it-Entwicklung, PriJfung und Anwendung. Soz. Prav..

[34] Williams B., Mancia G., Spiering W., Rosei E.A., Azizi M., Burnier M., Clement D., Coca A., De Simone G., Dominiczak A. (2018). 2018 practice guidelines for the management of arterial hypertension of the European society of cardiology and the European society of hypertension ESC/ESH task force for the management of arterial hypertension. J. Hypertens..

[35] Weisell R.C. (2002). Body mass index as an indicator of obesity. Asia Pac. J. Clin. Nutr..

[36] dos Santos J., Gouveia M.C., de Souza Júnior F.A., da Silva Rodrigues C.E., dos Santos J.M., de Oliveira A.J.S. (2018). Effect of a High-Intensity Interval Training Session on Post-Exercise Hypotension and Autonomic Cardiac Activity in Hypertensive Elderly Subjects. J. Exerc. Physiol. Online.

[37] Schimitt R.P., Carpes L.O., Domingues L.B., Tanaka H., Fuchs S.C., Ferrari R. (2020). Effects of a single bout of power exercise training on ambulatory blood pressure in older adults with hypertension: A randomized controlled crossover study. Complement. Ther. Med..

[38] Canuto P.M.d.B.C., Nogueira I.D.B., da Cunha E.S., Ferreira G.M.H., de Mendonça K.M.P.P., da Costa F.A., Nogueira P.A.d.M.S. (2011). Influence of resistance training performed at different intensities and same work volume over bp of elderly hypertensive female patients. Rev. Bras. Med. Esporte.

[39] Brandão Rondon M.U.P., Alves M.J.N.N., Braga A.M.F.W., Teixeira O.T.U.N., Barretto A.C.P., Krieger E.M., Negrão C.E. (2002). Postexercise blood pressure reduction in elderly hypertensive patients. J. Am. Coll. Cardiol..

[40] Vlachopoulos C., Aznaouridis K., Stefanadis C. (2010). Prediction of Cardiovascular Events and All-Cause Mortality with Arterial Stiffness. A Systematic Review and Meta-Analysis. J. Am. Coll. Cardiol..

[41] Wisløff U., Støylen A., Loennechen J.P., Bruvold M., Rognmo Ø., Haram P.M., Tjønna A.E., Helgerud J., Slørdahl S.A., Lee S.J. (2007). Superior cardiovascular effect of aerobic interval training versus moderate continuous training in heart failure patients: A randomized study. Circulation.

[42] Jeeva K., Bhattacharya P. (2018). Effect of body mass index on post-exercise hypotension in healthy adult males. Natl. J. Physiol. Pharm. Pharmacol..

[43] Pescatello L.S., Guidry M.A., Blanchard B., Kerr A., Taylor A.-L., Johnson A.N., Maresh C.M., Rodriguez N., Thompson P.D. (2004). Exercise intensity alters postexercise hypotension. J. Hypertens..

[44] Roth G.A., Mensah G.A., Johnson C.O., Addolorato G., Ammirati E., Baddour L.M., Barengo N.C., Beaton A., Benjamin E.J., Benziger C.P. (2020). Global Burden of Cardiovascular Diseases and Risk Factors, 1990–2019: Update from the GBD 2019 Study. J. Am. Coll. Cardiol..

[45] Wang K.L., Cheng H.M., Chuang S.Y., Spurgeon H.A., Ting C.T., Lakatta E.G., Yin F.C.P., Chou P., Chen C.H. (2009). Central or peripheral systolic or pulse pressure: Which best relates to target organs and future mortality?. J. Hypertens..

[46] Esformes J.I., Norman F., Sigley J., Birch K.M. (2006). The influence of menstrual cycle phase upon postexercise hypotension. Med. Sci. Sport. Exerc..

[47] Ketelhut S., Ketelhut R.G., Weisser B., Nigg C.R. (2022). Interval Training in Sports Medicine: Current Thoughts on an Old Idea. J. Clin. Med..

